# β_2_-AR blockade potentiates MEK1/2 inhibitor effect on HNSCC by regulating the Nrf2-mediated defense mechanism

**DOI:** 10.1038/s41419-020-03056-x

**Published:** 2020-10-13

**Authors:** Luigi Mele, Vitale Del Vecchio, Francesco Marampon, Tarik Regad, Sarah Wagner, Laura Mosca, Sabrina Bimonte, Aldo Giudice, Davide Liccardo, Claudia Prisco, Melanie Schwerdtfeger, Marcella La Noce, Virginia Tirino, Michele Caraglia, Gianpaolo Papaccio, Vincenzo Desiderio, Antonio Barbieri

**Affiliations:** 1 Department of Experimental Medicine, University of Campania “Luigi Vanvitelli” via L. Armanni 5, 80138 Naples, Italy; 2 Department of Radiotherapy, Policlinico Umberto I, “Sapienza” University of Rome, 00185 Rome, Italy; 3 The John van Geest Cancer Research Centre, School of Science and Technology, Nottingham Trent University, Clifton Lane, Nottingham, NG11 8NS UK; 4 Department of Precision Medicine, University of Campania “Luigi Vanvitelli”, Via De Crecchio, 16, 80138 Naples, Italy; 5 Division of Anesthesia and Pain Medicine, Istituto Nazionale Tumori-IRCCS-“Fondazione G. Pascale”, Via Mariano Semmola, 80131 Naples, Italy; 6 Epidemiology Unit, Istituto Nazionale Tumori “Fondazione G. Pascale”, IRCCS, Via Mariano Semmola, 80131 Naples, Italy; 7 Animal Facility, Istituto Nazionale Tumori-IRCCS-Fondazione “G. Pascale”, “Fondazione G. Pascale”, Via Mariano Semmola, 80131 Naples, Italy

**Keywords:** Targeted therapies, Oral cancer

## Abstract

The β2-Adrenergic receptor (β2-AR) is a G protein-coupled receptor (GPCR), involved in the development of many cancers, among which HNSCC. In this contest, β2-AR signaling interacts with different pathways, such as PI3K and MAPK, commonly activated by TK receptors. For this reason, TK blockade is one of the most adopted therapeutic strategies in HNSCC patients. In our study we investigated the effects of the β2-AR blocking in HNSCC cell lines, using the selective inhibitor ICI118,551 (ICI), in combination with the MAPK inhibitor U0126. We found that ICI leads to the blocking of p38 and NF-kB oncogenic pathways, strongly affecting also the ERK and PI3K pathways. Cotreatment with U0126 displays a synergic effect on cell viability and pathway alteration. Interestingly, we found that the β2-AR blockade affects Nrf2-Keap1 stability and its nuclear translocation leading to a drastic ROS increase and oxidative stress. Our results are confirmed by a TCGA dataset analysis, showing that NFE2L2 gene is commonly overexpressed in HNSC, and correlated with a lower survival rate. In our system, the PI3K pathway inhibition culminated in the blocking of pro-survival autophagy, a mechanism normally adopted by cancer cells to became less responsive to the therapies. The mTOR expression, commonly upregulated in HNSC, was reduced in patients with disease-recurrence. It is well known that mTOR has a strong autophagy inhibition effect, therefore its downregulation promoted pro-survival autophagy, with a related increase recurrence rate. Our findings highlight for the first time the key role of β2-AR and related pathway in HNSCC cell proliferation and drug resistance, proposing it as a valuable therapeutic molecular target.

## Introduction

The β2-Adrenergic receptor (β2-AR) is a G protein-coupled receptor (GPCR), which belongs to the superfamily of adrenergic receptors. They are activated by several catecholamines, in response to physiologic stimulations of the sympathetic nervous system (SNS), such as bronchodilation or smooth muscle relaxation. The activation of the β2-AR signaling pathway drives cell malignant development^1–3^.

Recent studies proved that α-AR (α1 and α2 subtypes) and β-AR (β1, β2, and β3 subtypes) are expressed in several neoplasias, such as hemangioma, melanoma, ovarian, prostate, lung, breast, liver, and head and neck cancers^4–7^.

β-AR activation leads to the synthesis of cyclic AMP (cAMP), through the guanine nucleotide-binding protein (Gαs), involved in the regulation of several cellular processes, including differentiation, proliferation, and apoptosis. Protein Kinase A (PKA) has a key role in the β-AR pathway through the activation of the β-AR kinase (BARK), which induces the transient desensitization of β-AR by β-arrestin, and activates the PI3K/Akt/mTOR and Src/Ras/MAPK pathways. A second major cAMP downstream signaling involves the guanine nucleotide exchange protein (EPAC), that was reported to regulate cell morphology, motility and secretion dynamics, through the activation of the Ras-related protein Rap-1A, which stimulates the downstream kinases B-Raf and the MAP/extracellular signal-regulated kinases 1/2 (ERK1/2). Several studies thoroughly described the ERK/MAPK pathway, that is mainly activated by tyrosine kinase receptors (RTK), such as EGFR, and that is over-activated in melanoma, colon, head and neck and breast (HER2+) cancers^8–10^. In the latter, the constitutive HER2− mediated activation of ERK induces an autocrine release of epinephrine, with positive feedback, that promotes cell growth^11^.

Targeted therapies with MAPK inhibitors impair the expression of several proteins involved in the control of cell differentiation, proliferation, and apoptosis. These therapeutic strategies are very effective for the treatment of several malignancies, such as colon, breast, lung^12,13^, and head and neck cancers.

The β2-AR activation induces a neo-angiogenic switch that drives prostate cancer progression from the low-grade pre-neoplastic stage to the high-grade malignant stage^14^. Other studies also demonstrated that β2-AR is involved in TKI (trastuzumab) resistance in breast cancer through the PI3K/AKT/mTOR signaling pathway^15^. Moreover, it has been demonstrated that EGFR inhibitor resistance in NSCLC (Non-Small-Cell Lung Carcinoma) correlates with the activation of the β2-AR pathway through an IL-6 dependent mechanism^16^.

The PI3K pathway components also regulate the REDOX balance in cancer cells by directly promoting the nuclear factor erythroid 2-related factor 2 (Nrf2)-mediated response. The Nrf2/Keap1 complex is a key effector in the cell-protective mechanisms against environmental changes, such as ROS (reactive oxygen species) or xenobiotics. Nrf2 is a transcription factor that can be blocked, in its cytoplasmic form, by Keap1. ROS increase drives the dissociation of Nrf2 from Keap1 and promotes its translocation to the nucleus, where it can activate the transcription of antioxidants Responsive Elements (ARE)^17^.

It was demonstrated that the MAPK/PI3K pathways crosstalk, promotes drug resistance autophagy-mediated^18,19^. This is a physiologic cell process that leads to the degradation/recycle of cytoplasmic cellular components such as damaged organelles and proteins, that can have cytoprotective or pro-apoptotic effects. Moreover, cancer autophagy has a dualistic role as it can synergize with drugs by increasing their cytotoxicity or antagonize their effect by promoting the degradation of molecular targets or proteins that are involved in the therapeutic process^20,21^.

Some studies showed that the autophagic flux increases in response to MAPK inhibitors and therefore, its blockade could be considered as a therapeutic option. Autophagy gene targeting strategies or inhibitors (chloroquine, 3-Methyladenine) can sensitize resistant cells to MAPKi (MAPK inhibitors) treatments^22^.

Based on this evidences, we investigated a new drug resistance mechanism, that involves the β2-AR and MAPK pathways, using the selective inhibitors ICI118,551 (ICI) and MEK1/2 (U0126), respectively. We found that there is a synergic effect of the two drugs, which leads to an increased ROS production via Nrf2 inhibition. In this setting, the increase in the autophagic flux can be considered as a protective mechanism, due to Nrf2 inhibition that leads to HNSCC cell death.

## Results

### β_2_-AR blockade impairs HNSCC cell viability and has a synergistic effect with the MEK1/2 inhibitor

According to the literature, we studied the mechanisms underlying HNSCC development and progression, especially those related to the effect of the selective inhibition of the β2-AR in HNSCC cell lines UMSCC103 and CAL-33.

**Fig. 1:**
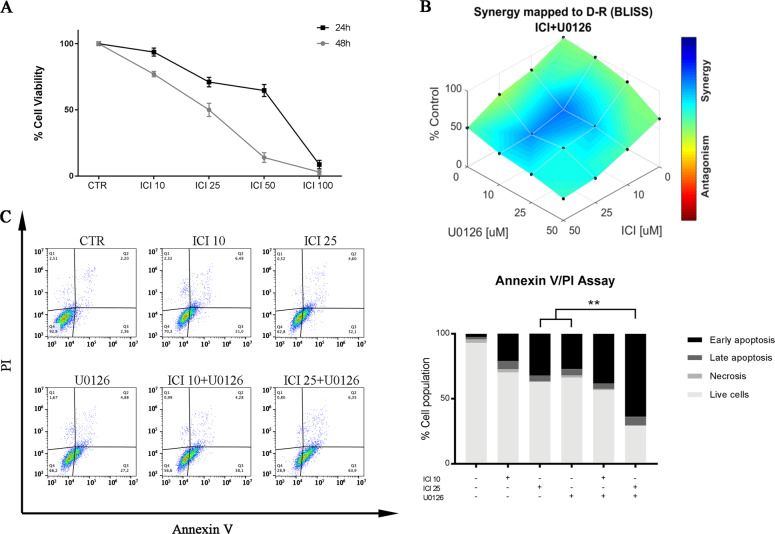
β2-AR and MEK1/2 inhibitors increase cell death by apoptosis, in a synergistic way. **a** Viability assay on UMSCC 103 treated with ICI at 24 h and 48 h. The β2-AR inhibitor IC50 was ~40 μM at 24 h and ~25 μM at 48 h. **b** The values are represented in the combination index plot in function of ICI/U0126 tested concentrations. The drugs show a significant synergism in two combinations (10 μM ICI + 10 μM U0126 and 25 μM ICI + 10 μM U0126). **c** Annexin V/PI flow cytometry analysis to determine apoptosis in ICI and U0126-treated cells. Drug combination promotes a strong apoptosis effect, on UMSCC 103, compared to the single treatments. Annexin V/PI assays are also shown in the histograms. (**P ≤ 0.01).

The viability of UMSCC103 cells was significantly reduced and in a dose and time-dependent manner after 48 h of treatment with 25 μM of ICI (Fig. 1a). We selected the IC50 and the IC25 (10 μM) for further studies.

Some studies described the role of the MAPK pathway in cancer resistance to targeted therapy^15,16^; therefore, we also investigated the role of this pathway in the UMSCC103 cell line using the MEK1/2 inhibitor. Several combinations of ICI and U0126 were tested to assess cell viability and to determine if there is a synergistic effect at different concentrations (Fig. 1b). Similar results have been obtained using CAL-33 cell line (Fig. S1A-B)

Using an apoptosis assay, based on Annexin V/PI staining, we observed that the drugs alone increased the levels of apoptosis and without any effect on cell necrosis. Interestingly, a synergistic effect on UMSCC103 and CAL33 cells apoptosis (apoptosis 70,25%) when ICI and U0126 were combined (Fig. 1c, S1C).

### β2-AR inhibition regulates the crosstalk between ERK and PI3K pathways

**Fig. 2:**
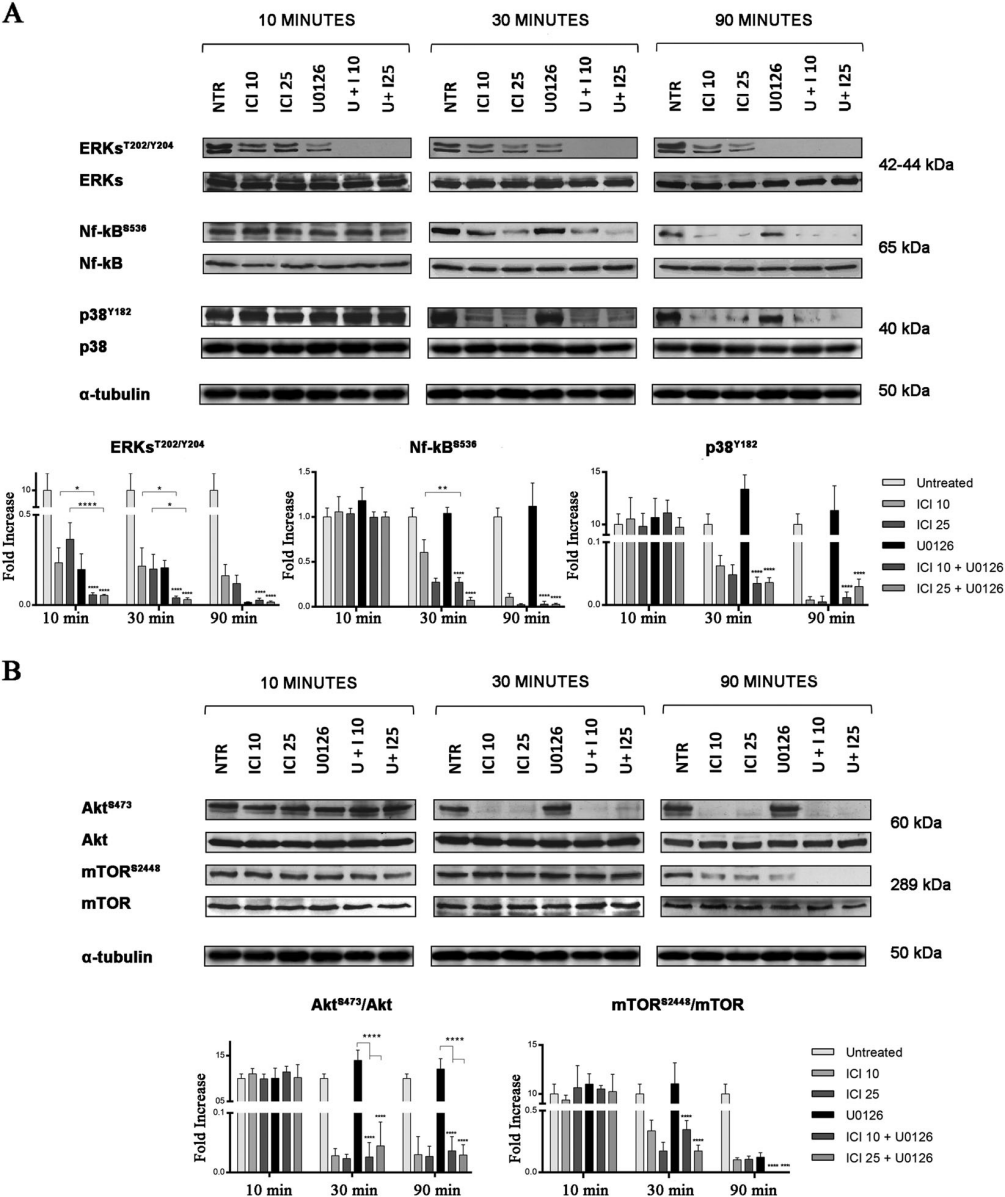
β2-AR and MEK1/2 inhibitors impair MAPK and PI3K pathways. **a** Immunoblot for ERK/ERK^T202/Y2014^, Nf-kB/Nf-kB^S536^, and p38/p38^Y182^ (below band densitometries); β2-AR blocking increases U0126 inhibition of ERK phosphorylation at a short time, the same trend is observed in Nf-kB. P38 phosphorylation is strongly reduced only over ICI treatment. **b** Immunoblot for mTOR/mTOR^S2448^ and Akt/AKT^S473^ (below band densitometries); the Akt ratio between phosphorylated and non-phosphorylated forms decreases over ICI treatment, while U0126 has no effect; on the other hand, it increases ICI inhibition of mTOR phosphorylation (*P ≤ 0.05, **P ≤ 0.01, ****P ≤ 0.0001).

The β2-AR/cAMP/PKA axis is directly involved in cancer development^7^ through the activation of other signaling molecules, among which, the mitogen-activated protein kinases (MAPKs) pathway^7^. In our model, we analyzed the role of the ERK/p38/NF-kB axis, which is commonly upregulated in HNSCC patients^23^. We found that short-time treatments (10 min.) of UMSCC103 cells with either ICI or U0126 significantly increase ERK phosphorylation. Moreover, the combination of the two drugs had a stronger effect on ERK phosphorylation (Fig. 2a). Subsequently, we also investigated p38 and NF-kB, which are two of the most involved molecules in the ERK pathway. We observed a marked similarity in their activation. The phosphorylations of p38 and NF-kB were significantly reduced with ICI alone or in combination with U0126, which otherwise was not able to reproduce this effect when solely used (Fig. 2a). These results suggest that the NF-kB and p38 activities were not directly regulated by MEK1/2, but probably through alternative signaling.

Several studies described the cross-talk between β2-AR and other molecular axes in cancer^15^. In particular, we focused on the role of the PI3K and the well-characterized RAS/MEK/ERK signaling in the development and progression of HNSCC. Several clinical trials are currently ongoing in patients that are treated with PI3K and/or mTOR inhibitors, and in combination with chemotherapeutic agents or an anti-EGFR mAB^24^. Moreover, recent studies demonstrated alternative molecular pathways, that are promoted by β2-AR, and that involves the G_i_, but not G_s_ protein, and which activate the downstream PI3K signaling pathway^25^.

We investigated the PI3K/AKT/mTOR pathway in the UMSCC103, following treatment with ICI, to determine the effect of β2-AR inhibition in pathways cross-talk blocking. We found that ICI reduced the activation of both AKT and mTOR and this effect was more important when combined with U0126 and after 90 min. Conversely, the treatment with U0126 alone did not affect AKT phosphorylation; while, mTOR phosphorylation decreased to ICI treatment levels after 90 min (Fig. 2b).

### β2-AR antagonist increases oxidative stress via NFR2 pathway blockade

**Fig. 3:**
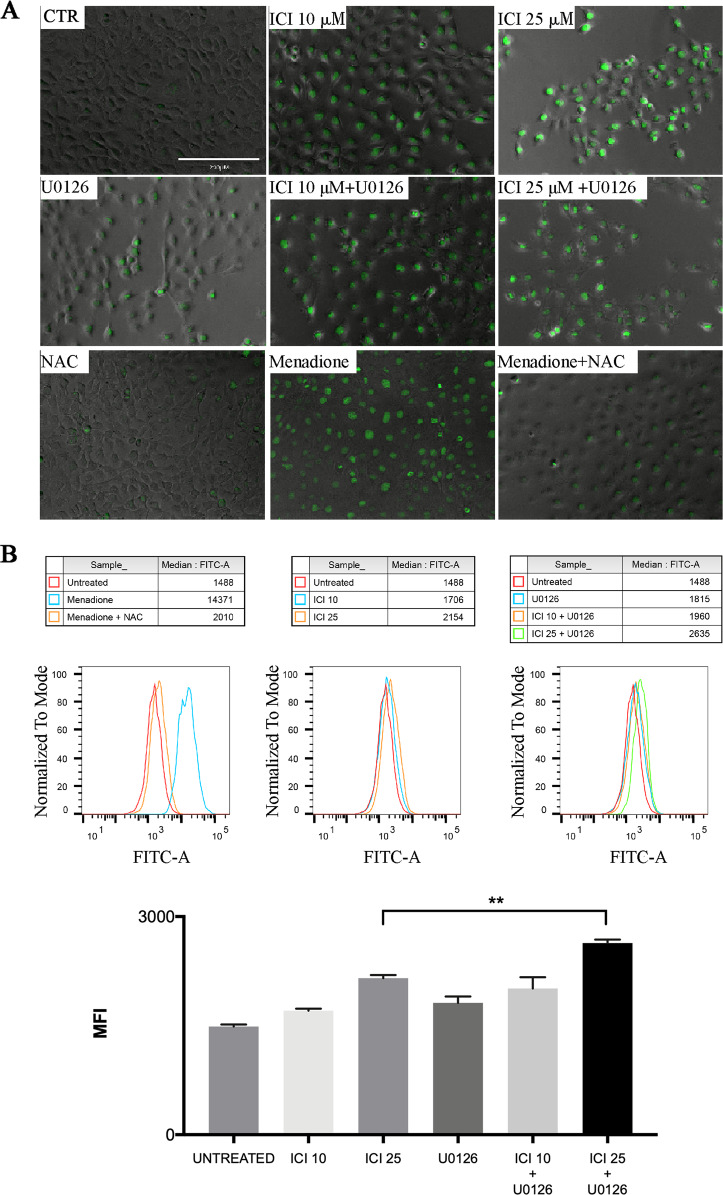
β2-AR inhibition induces oxidative stress blocking NRF2 nuclear translocation. **a**–**b** IF and Flow cytometry with CellROX (for oxidative stress determination), 24 h posttreatment (menadione is used as a positive control; N-acetylcysteine (NAC) is used as ROS scavenger). ICI treatment strongly increases ROS production, instead of U0126 which has a milder effect. The Flow Cytometry experiments have been performed in triplicates and the statistical analysis confirmed the significance of the results (**P ≤ 0.01).

**Fig. 4:**
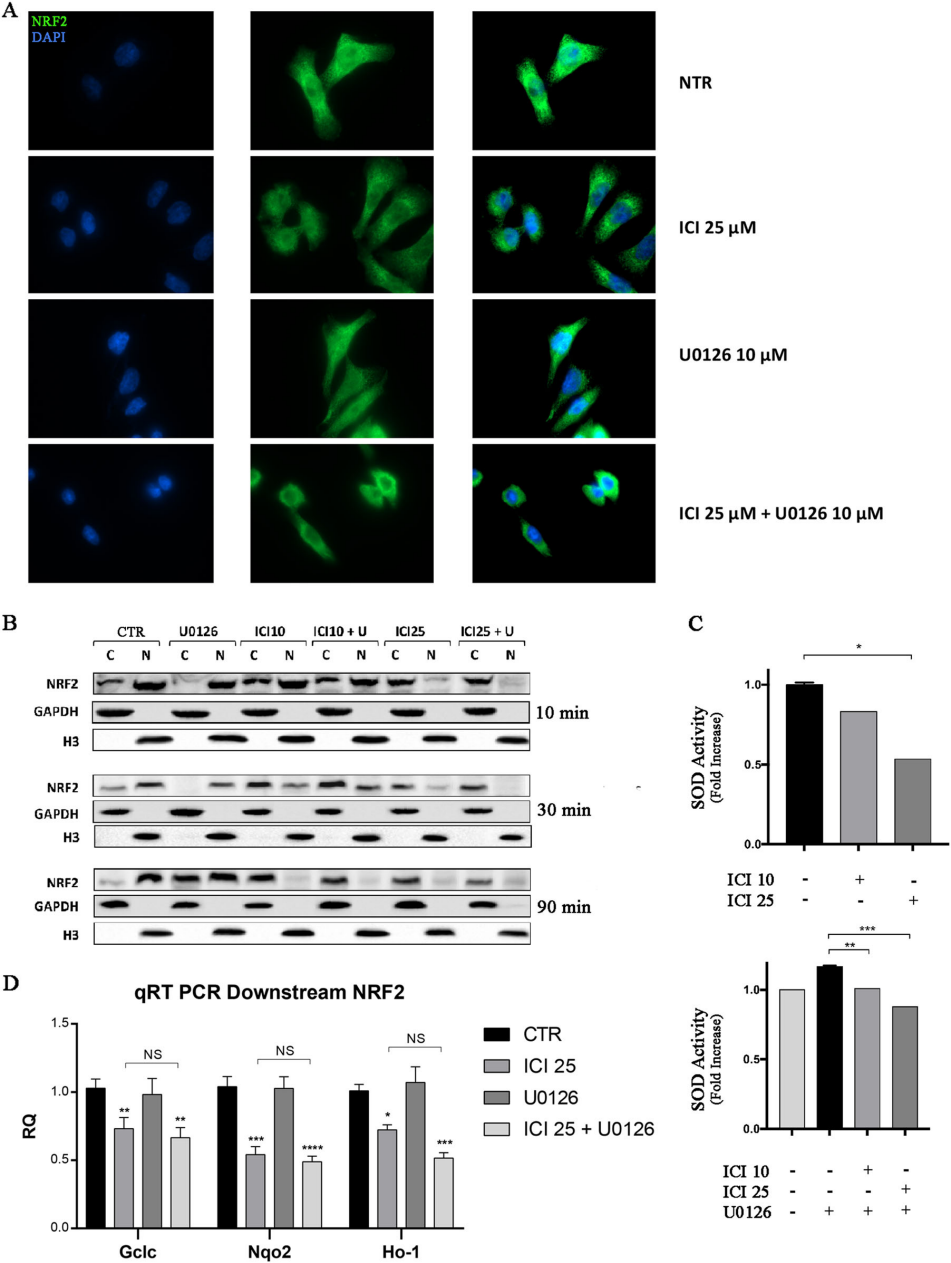
NRF2 perturbation. **a** IF of NRF2 localization at 90 min. ICI treatment promotes the cytoplasmatic block of NRF2 **b** Immunoblot for cytoplasmatic and nuclear NRF2. ICI treatment promotes the cytoplasmatic block of NRF2; otherwise, U0126 has a low opposite effect after 30 min. **c** SOD activity assay. ICI reduces SOD activity in a dose-dependent manner (*P ≤ 0.05). MEK 1/2 inhibitor mildly increases it, ICI reverts its effect (**P ≤ 0.01, ***P ≤ 0.001). **d** qRT PCR of NRF2 downstream. ICI treatment reduced the expression of all NRF2 downstream genes (*P ≤ 0.05, **P ≤ 0.01, ***P ≤ 0.001).

The increased levels of free radicals and peroxides lead to cellular oxidative stress, due to an unbalanced metabolism or cellular insults. In this condition, the cells activate several processes to readily detoxify these intermediates and repair the resulting damages^17^. To further investigate the biological mechanisms underlying the cytotoxic effect of the drugs, we analyzed the levels of ROS after treatments of UMSCC103 and CAL33 cells with ICI, U0126, or their combination and in a dose-dependent manner (Fig. 3a–b, S2B). In this context, we also investigated Nrf2 translocation into the nucleus, and that is physiologically inhibited by Keap1. We observed that the treatment with both drugs had a stronger effect on the activation of Nrf2; however, treatment ICI single treatments had significant time and dose-dependent inhibition of Nrf2 translocation after 10 and 30 min. Conversely, a longer inhibition time (90 min.) showed an enhancement of nuclear and cytoplasmic Nrf2 levels, probably due to ROS accumulation (Fig. 4b). We confirmed this result also by immunofluorescence at 90 min. of treatment (Fig. 4a).

These results demonstrate that Nrf2 blockade, mediated by the β_2_-AR antagonist, leads to the inhibition of the super-oxide-dismutase (SOD). SOD activity was significantly lower in the samples treated with ICI at 25 μM (*P* ≤ 0.05). Conversely, the treatment with U0126 led to an increment in SOD activity, that was reverted by ICI in the combined treatment in a dose-dependent fashion (*P* ≤ 0.001*)* (Fig. 4c). Moreover, the functional block of the NRF2 has been confirmed by evaluating the expression of its downstream genes Gclc, Nqo2, and Ho-1. We observed for all these genes that the treatment with ICI (90 min.) strongly reduced their expression (Fig. 4d).

### Autophagic flux impairment is driven by β_2_-AR inhibition

**Fig. 5:**
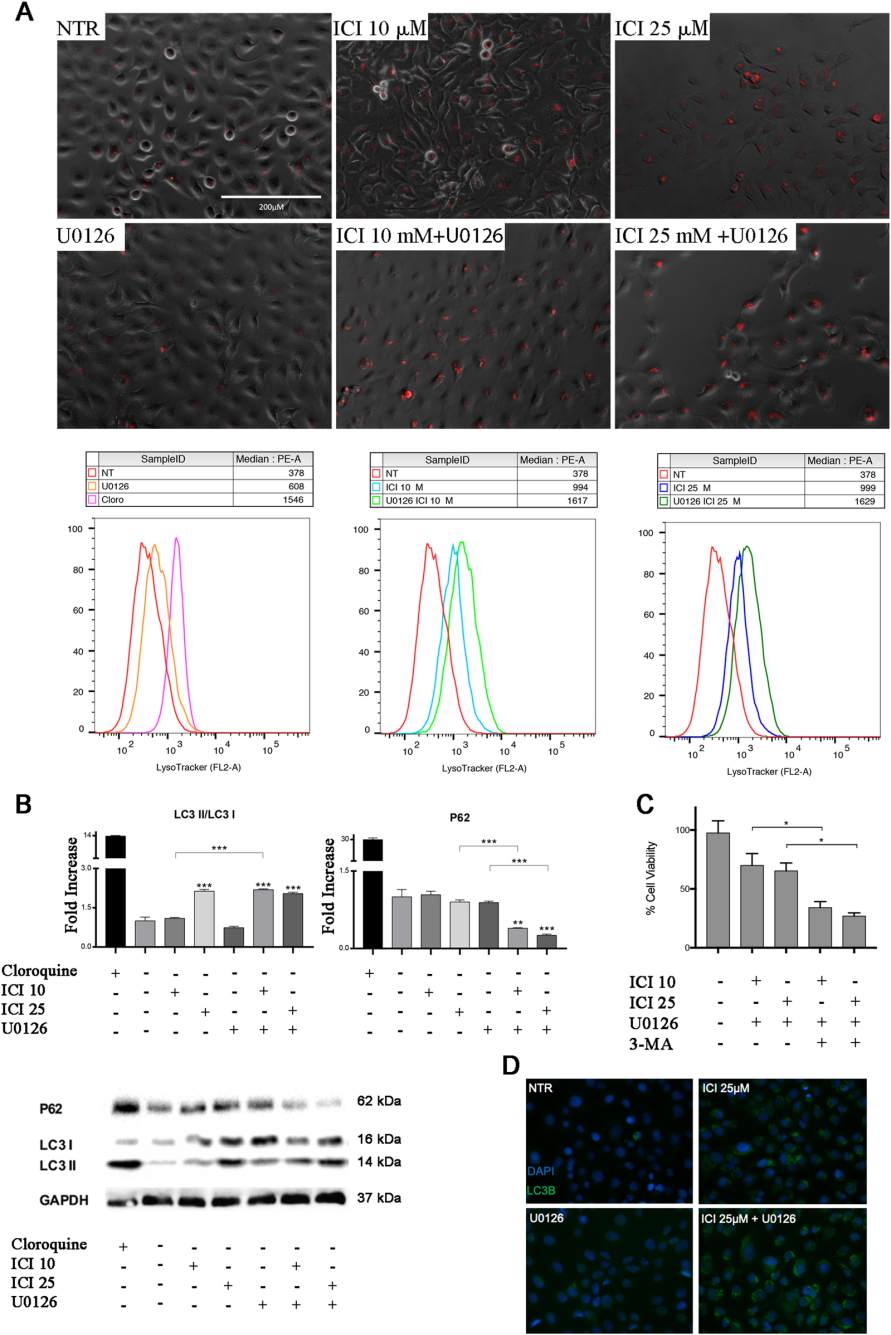
β2-AR inhibition induces autophagy. **a** IF and flow cytometry for LysoTracker. ICI treatment increases the number and size of lysosomes on UMSCC 103, after 24 h. Flow cytometry histograms for the LysoTracker are shown in the graphs. **b** Immunoblot for LC3B and p62; band densitometry in ICI and U0126 treated cells, 24 h after treatment. LC3BII increases in ICI alone and ICI/U0126 samples while p62 decreases. **c** Viability assay on UMSCC103 treated with ICI and U0126 plus 3-MA. 3-MA strongly increases the cytotoxicity of the drug combination (*P ≤ 0.05, **P ≤ 0.01, ***P ≤ 0.001). **d** IF for LC3B, ICI treatment increased the number of autophagosomes.

The PI3K pathway is a well-known regulator of the autophagic flux. In our model, we found lower phosphorylation levels of AKT and mTOR, a direct autophagy flux inhibitor. Autophagy is a self-degradative process that is activated in several conditions by the cell, and that mainly regulates the energetic metabolism during development, or after cell insults^22^. This process is mediated by the activation of survival mechanisms, that lead to cell materials recycling, misfolded/aggregated protein degradation, and pathogen elimination. We investigated this process in UMSCC103 cells using fluorescence microscopy, on LysoTracker stained samples treated with β2-AR and MEK1/2 inhibitors. In our model, treatments with ICI or U0126 alone did not significantly affect the auto-phagosome number and size^20^; while, the combination of the drugs led to a stronger effect (Fig. 5a, S2A). Moreover, we confirmed the effect on autophagosome by LC3B immunofluorescence (Fig. 5d).

To further investigate the molecular regulation of the autophagy flux in our model, we analyzed the expression of p62, an autophagy clearance marker, involved in the turnover of the proteins that constitute the autophagosome scaffold. We observed a significant decrease in p62 level of expression when a combination treatment with ICI and U0126 was used, and that resulted in an increased rate of autophagosome degradation. Meanwhile, we analyzed the expression of LC3 II/LC3 I, a direct interactor of p62 and considered a marker of the early autophagosome scaffold vesicles building. In our case, the treatment with the combination drugs led to a significant enhancement of LC3 II/LC3 I levels, which correlated with increased number and size of autophagosome vesicles. The same trend was observed after treatment with ICI at the concentration of 25 μM. These results demonstrate an autophagy flux activation that is driven by β2-AR inhibition, and that is increased in combination with the MEK1/2 inhibitor (Fig. 5b). To investigate the role of autophagy in our model, we analyzed UMSCC103 viability when co-treating them with the autophagy flux inhibitor 3-methyladenine (3-MA). We observed that 3-MA significantly reduced cell viability, after treatment with ICI (*P* ≤ 0.05), U0126 (*P* ≤ 0.05), or a combination of both (*P* ≤ 0.05). This experiment suggests that, in UMSCC103 cells, β2-AR and MEK1/2 blockade induces the activation of autophagy as a protective mechanism and in response to cell insults mediated by the drugs (Fig. 5c).

### Clinical screening of ADRB2 expression and associated genes in HNSCC patients

**Fig. 6:**
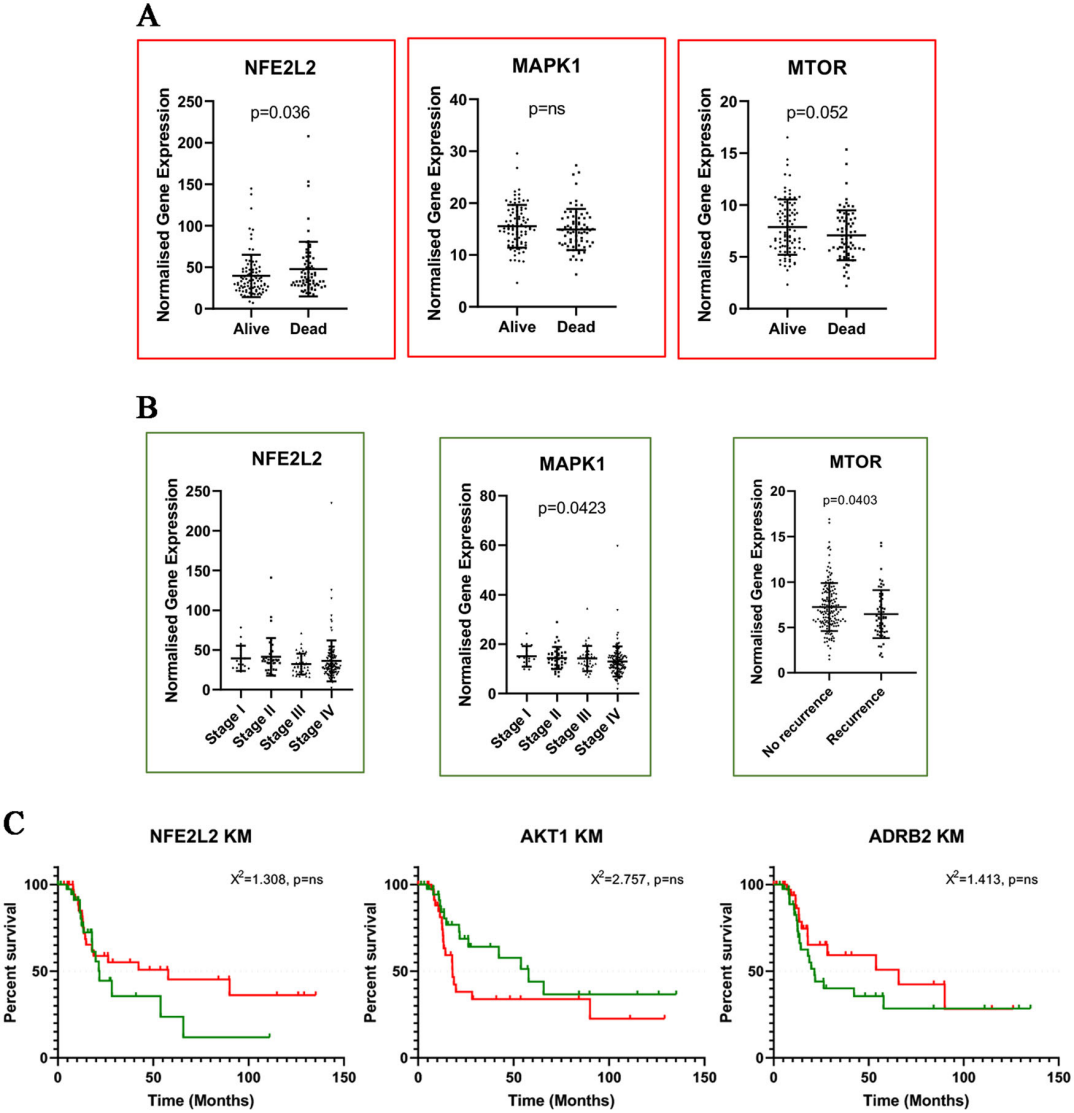
β2-AR expression is correlated with MAPK, PI3K, and ROS metabolism molecular effectors. **a** In silico TCGA data set analysis on HNSC patients showed a strong correlation between NFE2L2 expression and survival rate. Lower not significant mTOR levels are also reported. **b** Scatter plot presenting the normalized genes expression within β2-AR + expression different disease stages. The same genes were evaluated for disease recurrence and here only MTOR (panel **c**) has shown a significantly lower expression in patients with disease recurrence. *P*-values below 0.05 were considered to be significant Analyses were performed using GraphPad Prism 8.

To identify a potential clinical association between NFE2L2, MAPK1, MTOR, AKT1, and ADRB2 in HNSCC patients, an in-silico analysis of the TCGA-HNSC dataset was performed. Initially, a comparison of gene expression in patients with and without survival was performed. Here, the expression of NFE2L2 (Fig. 6a) was shown to be significantly increased in patients without survival; whereas, no significant differences could be found in MAPK1 (Fig. 6a). The difference in the expression of mTOR (Fig. 5a) across these two patient groups was not significant but presented a visible trend for an association between higher gene expression and increased survival.

To study a closer relationship of selected genes (NFE2L2, MAPK1, and MTOR) in correlation with ADRB2, the patients with an above-median expression of ADRB2 were selected for further analyses. MAPK1 was the only gene that presented a significant reduction in expression between Stage IV and stage I of disease (*p* = 0.0423) (Fig. 6b). The same genes were evaluated for disease recurrence and we found that mTOR only (Fig. 6c) had significantly lower expression in patients with disease recurrence. No significant differences were observed in MAPK1 and NFE2L2 (Fig. 6c).

## Discussion

β2-AR is a GPCR involved in the transmission of intracellular signals, in response to oxidative stress or hormone stress-mediated pathways. The activation of β2-AR can regulate several processes that are involved in cancer initiation and progression, inflammation, neo-angiogenesis, apoptosis and anoikis, cell motility, immune cell response, and epithelial-mesenchymal transition. β2-AR signaling activates several pathways, including the PI3K/AKT/mTOR and/or RAF-MEK-ERK pathways^7^. It has been demonstrated that this receptor is overexpressed in several types of HNSCC and correlates with worse prognosis^26^. Considering the status of β2-AR in HNSCC, we decided to investigate the role of this receptor in the resistance to targeted therapy resistance, which is mediated by the above downstream pathways.

The low survival rate of HNSCC patients is due to delayed diagnosis, recurrence, and drug resistance. New approaches have been developed to overcome these limitations by promoting new therapeutic strategies against more-specific molecular targets^27^. Many studies showed promising results when using several compounds that target the MAPK pathway components. In the clinic, cetuximab is the most adopted targeted therapy for HNSCC. This drug directly binds the extracellular domain of EGFR and inactivates downstream ERK1/2 and/or PI3K/Akt signaling, consequently inhibiting cell cycle progression, angiogenesis, and metastasis^28^.

Our study aimed at characterizing β2-AR associated pathways, at the phenotype and molecular levels and investigate their roles in HNSCC drug resistance. For this, we inhibited β2-AR and MEK1/2 with selective inhibitors and we found, that the inhibition of β2-AR and MEK1/2 reduced cell viability by inactivating apoptosis. This effect confirms that UMSCC103 cell proliferation is impaired by EGFR downstream blockade, but also by alternative pathways involving the β2-AR signaling. Surprisingly, the combined treatment had a synergistic effect, compared to the single treatments, and in a concentration-dependent manner.

To further understand the molecular mechanisms underlying drugs cytotoxicity, we analyzed the expression and the activation levels of the molecular factors that are involved in β2-AR and EGFR pathways. The ERK pathway is significantly inhibited in UMSCC103 cells after a long period of treatment with U0126, and the inhibition was less significant following treatment with ICI. Interestingly, the combination of 2 drugs induced a blockade of ERK phosphorylation after only 10 min. of treatment. In this context, we confirmed that β2-AR blocking affected cancer proliferation, probably through its interaction with other pathways, such as the ERK pathway^29^. Therefore, ICI can potentiate U0126-induced ERK inhibition and reducing related cytotoxicity. To clarify the role of β2-AR signaling and associated pathways, we analyzed the activation of the downstream molecular effectors that were mainly involved in cancer. Therefore, we focused on the p38 kinase that is involved in several cancer biological mechanisms, such as cell survival, differentiation, migration, drug resistance, neo-angiogenesis, and inflammation (IL-6 and TNF-α)^30^. The p38 kinase regulates cytokines expression by modulating the activity of several transcription factors, among which the Nuclear Factor kappa-B (NF-kB), that is mostly involved in cancer progression and relapse. The regulation of the p38 gene is also promoted at the post-transcriptional level through mechanisms that affect mRNA stability and protein translation^30,31^. NF-kB can be considered a crossroads between β2-AR and MAPK pathways^25,32^, therefore, we hypothesized that it could be impaired by the drugs used in the study. We found that the selective blockade of the β2-AR signaling inhibits the phosphorylation of both p38 and NF-kB; while, U0126 did not affect their activation.

**Fig. 7:**
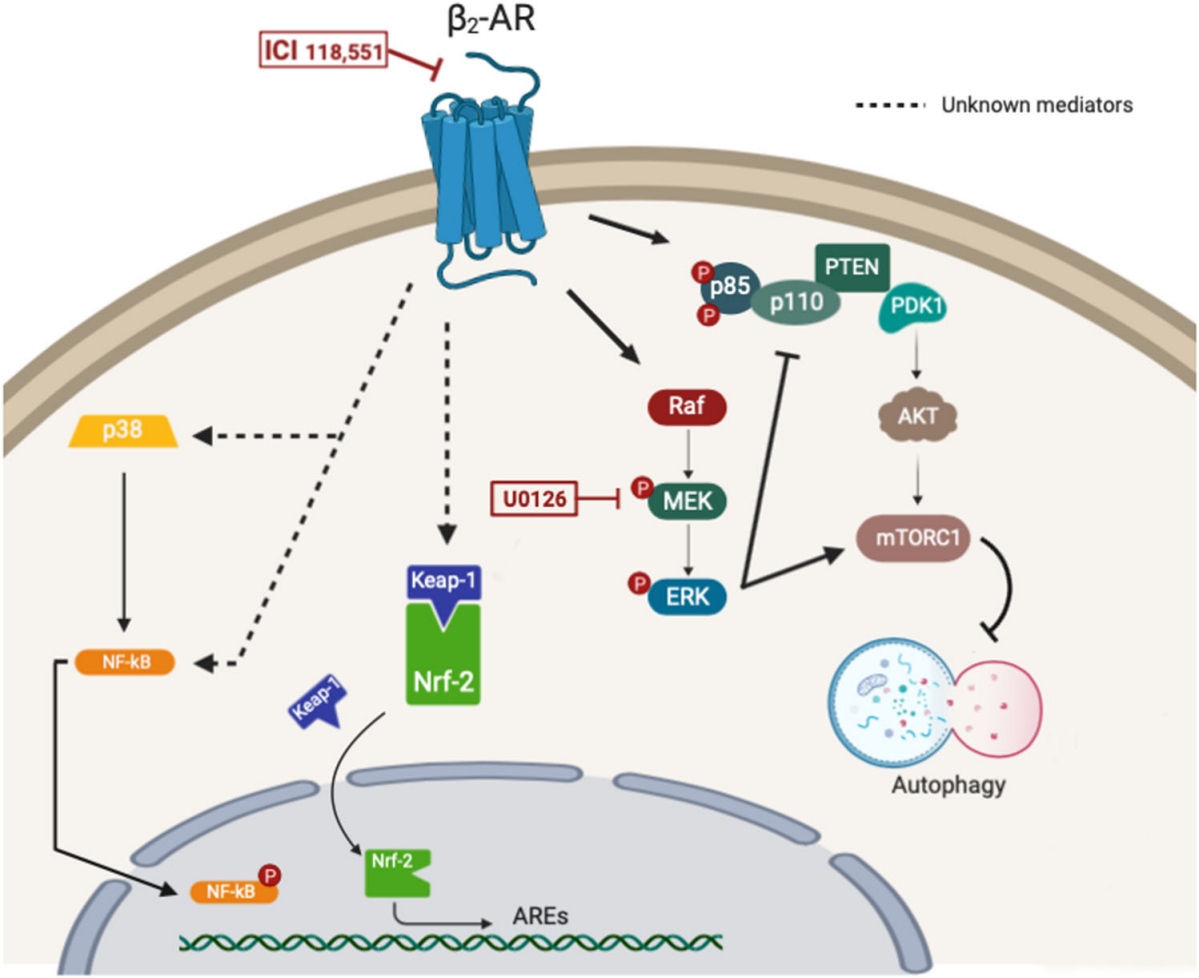
Inhibition of β_2_-AR block Nrf-2, p38, and NF-kB activity and potentiate MEK inhibitor effect on cancer. Graphical representation of pathways cross-talk involving β2-AR inhibition.

Several studies demonstrated that the PI3K/Akt/mTOR axis is an alternative downstream signaling pathway of β2-AR^25^. Our results confirmed this mechanism, as ICI induced a reduction in Akt activation, after 30 min. of treatment; while, U0126 had no effect. As expected, mTOR phosphorylation was also reduced after 90 min. of treatment with the β2-AR inhibitor. Interestingly, following treatment with the MEK1/2 inhibitor, we observed a similar effect. It is well known that Akt phosphorylation is linked to the downstream activation of mTOR; however, we observed a dualistic role of ERK in the regulation of the PI3K/Akt/mTOR pathway. According to literature^33^, our results confirmed that ERK can induce Akt inhibition and mTOR activation. In this way, we can make sense of the late synergic effect of the drugs on mTOR inhibition. Therefore, our results suggest a new signaling model, where both β2-AR and MAPK collaborate in ERK 1/2 and mTOR increased-activations. Indeed, our inhibitors showed a synergistic effect that was specific for these two kinases (Fig. 7).

Moreover, β2-AR also activates p38 and AKT, with a downstream involvement of NF-kB and alternative pathways. These effectors, together with mTOR and ERK, are implicated in the regulation of oxidative stress and autophagy^34–36^. In particular, the analysis of oxidative stress levels highlighted a dose-dependent increase in ROS production after ICI treatment of UMSCC103 cells and a lower effect in cells treated with U0126. The combination of drugs leads to a higher ROS production, confirming, that the blockade of both pathways increased the above effect when compared to single treatments. It is well-known that the PI3K/Akt/mTOR and MAPK axis can directly affect the nuclear translocation of Nrf2, the master regulator of the oxidative stress response^37^. Interestingly, the selective blockade of β2-AR leads to the retention of Nrf2 in the cytoplasm, promoting an increased ROS production due to the inhibition of the antioxidant cell response, and the related activation of the apoptotic program. This evidences suggest that the selective targeting of these important pathways in cancer could be an important strategy to reduce drug resistance in cancer cells and improve anticancer effects as a result of ROS production.

The regulation of Nrf2 has a key role in cancer cell survival, due to its downstream transcriptional activation of several ROS metabolism effectors. In our context, MEK1/2 inhibition mildly regains Nrf2 nuclear translocation, with the related upregulation of the SOD activity. Conversely, ICI blocked Nrf2 translocation for a short time, which is also confirmed by the functional assay on the SOD activity. These results suggest that the ROS increase, which was observed in the treated samples, is probably due to two distinct effects: U0126 mediated ROS increase, and ICI inhibition of the oxidative stress defense.

Based on our results about mTOR activation and its involvement in autophagy regulation^38^, we investigated this cellular mechanism, while also considering pieces of evidence on its key role in drug resistance^39^. Lysosomes formation was increased in all samples and particularly after co-treatment, which suggests the perturbation of autophagy in UMSCC103 cells. The vesicular flux analysis has been performed by quantifying the autophagosomes scaffolding, which correlated with the LC3 lipidation mechanism and p62 mediated degradation. Our results showed, that U0126 alone, did not impact on autophagy; while, ICI mildly promoted the autophagosome formation, especially at treatment concentration of 25 μM. Furthermore, the drug combination strongly increased the flux rate, probably due to the described dualistic role of ICI and U0126 in the regulation of interconnected pathways. We also investigated the effect of the activation of this mechanism in the pro-apoptotic/pro-survival balance. We observed that the treatment of UMSCC103 cells with the autophagosome formation inhibitor 3-MA, increased the effect of ICI and U0126 combination. This event suggests that autophagy can be considered as a protective mechanism that is employed by cancer cells to overcame drug cytotoxicity.

Several TCGA dataset studies showed that the NFE2L2 gene is commonly overexpressed in cancer tissues^40^ and particularly in HNSCC^41^. In our study, we found a strong correlation between the overexpression of this gene and a lower survival rate. Moreover, we found that mTOR, which is commonly upregulated in HNSC^42^, was expressed at low levels in patients with disease-recurrence. mTOR is an autophagy inhibitor and its downregulation in recurrent patients is potentially due to the protective role of autophagy that is reported in our study.

## Conclusion

In conclusion, our findings demonstrate that the inhibition of β2-AR and MAPK pathways has a synergistic effect on UMSCC cell viability. In our model, the β2-AR blockade affects PI3K/Akt/mTOR, p38, and NFkB activations; while, the MEK1/2 inhibitor affects ERK phosphorylation. The combination of the 2 drugs amplifies their effects on mTOR and ERK.

We have also found that the drug combination strongly increases the oxidative stress in UMSCC103 cells through the regulation of Nrf2 nuclear translocation. Moreover, β2-AR/MEK1/2 inhibition enhances the autophagic flux rate and its blockade increases their cytotoxicity, which highlights a protective effect autophagy-mediated.

We show, through the analysis of patient data sets, that ADRB2 expression correlates with the expression of MAPK1 and mTOR.

This evidences provide new insights for the treatment of HNSCC.

## Material and methods

### Chemicals, cell culture, and in vitro treatment

All chemicals were purchased from Sigma-Aldrich (St. Louis, USA) unless otherwise specified. Selective inhibitors of β2-AR (ICI118,551) and MEK1/2 (U0126) were obtained from Tocris Bioscience (Bristol, United Kingdom). 3-Methyladenine were purchased from Merck (Darmstadt, Germany).

UMSCC103 (OSCC cell line) used in this study was established at the University of Michigan under a protocol approved by the Institutional Review Board Office under the university’s regulations and described here15. The human head and neck cancer cell lines Cal-33 (oral squamous cancer cell lines) were obtained from the American Type Culture Collection (ATCC, Manassas, VA). Cells were cultured in DMEM and RPMI (Gibco, NY, USA) supplemented with 2 mM glutamine, 100 IU/mL penicillin, 100 μg/mL streptomycin (Invitrogen, Carlsbad, CA), and 10% heat-inactivated fetal bovine serum (FBS) (Gibco, NY, USA) at 37 °C in a humidified atmosphere under 5% CO2. The cell line was kept mycoplasma-free; checking was performed every three months.

### Cell viability assay

Cell viability was measured by the colorimetric 3-(4,5-dimethyl-2-thiazolyl)-2,5-diphenyltetrazolium bromide (MTT) assay. Cells were seeded in 96-well plates at a density of 10^4^ cells per well, then they were treated with 100 μL of 1 mg/mL MTT (Sigma) in DMEM medium containing 10% fetal bovine serum for 4 h at 37 °C. The medium was then replaced with 200 μL of DMSO and shaken for 15 min, then absorbance at 540 nm was measured using a microplate ELISA reader with DMSO used as the blank. To quantify the synergistic or antagonist effect of the drugs combinations, Combenefit® software was used^42^.

### CellRoX Assay

Cells were plated on glass-bottom 35-mm MatTek dishes and treated with ICI and/or U0126 for 24 h and 100 μM menadione for 1 h at 37 °C. A quantity of 50 μM N-acetylcysteine was added to menadione-treated wells. The cells were then stained with 5 μM CellROX green reagent by adding the probe to the complete media and incubating at 37 °C for 30 min. The cells were then washed with PBS and then imaged on a fluorescence microscope EVOS FL Cell Imaging System (Thermo Scientific, Rockford, USA). N-acetylcysteine treatment inhibited the fluorescent signal induced by menadione, confirming that the signal was specifically produced by ROS increase^43,44^.

### Live-Fluorescence staining

To stain lysosomes, cells were treated with the drugs, as explained before, and with Chloroquine (CQ) 100 µM for 2 h before staining with 60 nM LysoTracker (Thermo Fisher Scientific, USA) and incubation for 45 min at 37 °C.

### FACS analysis

Apoptosis (Annexin V apoptosis detection kit, BD biosciences), CellROX assay (Thermo Fisher Scientific, USA), LysoTracker assay (Thermo Fisher Scientific, USA), were performed according to the manufacturer’s instructions. Cells were analyzed with a FACSAria III (BD Biosciences, San Jose, CA) or a BD Accuri Cytometer (BD Biosciences, San Jose, CA). Data were analyzed by FlowJo V10 software (FlowJo LLC, USA).

### SOD enzymatic assay

SOD activity was measured by SOD Assay Kit WST, obtained from Dojindo, (Kunamoto, Japan). UMSCC103 cells untreated or treated with the drugs, were homogenized in cold PBS, followed by centrifugation at 8000×*g* for 10 min to remove insoluble materials. SOD activity was determined by analysis of SOD-dependent reduction of ROS, which leads to the conversion of WST-1 (water-soluble tetrazolium salt) in a water-soluble formazan dye (absorbance at 450 nm), linearly related to the SOD activity.

### Immunoblot analysis

Cells were lysed in 2% SDS containing 2 mM phenyl-methyl sulphonyl fluoride (PMSF) (Sigma), 10 μg/ml antipain, leupeptin and trypsin inhibitor, 10 mM sodium fluoride and 1 mM sodium orthovanadate (all from Sigma) and sonicated for 30 s. Proteins of whole-cell lysates were assessed using the Lowry method and equal amounts were separated on SDS-PAGE. The proteins were transferred to a nitrocellulose membrane (Schleicher and Schuell, BioScience GmbH, Germany) by electroblotting. The balance of total protein levels was confirmed by staining the membranes with Ponceau S (Sigma). Immunoblottings were performed with the following antibodies: anti-ERK2 (C-14, positive also for ERK1), anti-phospho-ERKs (E-4), anti-phospho-Akt1 (5.Ser 473), anti-Akt1 (G5), anti-phospho-p38 (E1), anti-p38 (A1F7), anti-phospho-mTOR (59.Ser 2448), anti-mTOR (30), and α-tubulin (B-7) (all from Santa Cruz Biotechnology, Santa Cruz CA); anti-LC3B (from Abcam, Cambridge UK).

Peroxidase-conjugate anti-mouse or anti-rabbit IgG (Amersham-Pharmacia Biotech, UK, or Santa Cruz) were used for enhanced chemiluminescence (ECL) detection.

### RNA isolation and qRT-PCR

Total RNA was isolated by RNeasy Mini Kit (Qiagen) according to manufacturer’s instructions, RNA was treated with DNase (Promega, Milan, Italy) to exclude DNA contamination and 1 μg total RNA reverse-transcribed using VILO SuperScript (Invitrogen, Monza, Italy). Gene expression assays were performed on a StepOne Thermocycler (Applied Biosystems, Monza, Italy) and the amplifications carried out using SYBR Green PCR Master Mix (Applied Biosystems, Monza, Italy). The reaction conditions were as follows: 95 °C for 15 min, followed by 40 cycles of three steps consisting of denaturation at 94 °C for 15 s, primer annealing at 60 °C for 30 s, and primer extension at 72 °C for 30 s. A melting curve analysis was performed from 70 °C to 95 °C in 0.3 °C intervals. Each sample was performed in triplicate. Glyceraldehyde 3-phosphate dehydrogenase (GAPDH) was used to normalize for differences in RNA input. Primers sequences are reported in Table 1.

**Table 1:** Sequences of primers used.

Gclc	Forward Primer	GGAAGTGGATGTGGACACCAGA
	Reverse Primer	GCTTGTAGTCAGGATGGTTTGCG
Nqo2	Forward Primer	GTATGCCATGAACCTTGAGCCG
	Reverse Primer	GCTCATCAGTGATGTCGCTAGC
Ho-1	Forward Primer	CCAGGCAGAGAATGCTGAGTTC
	Reverse Primer	AAGACTGGGCTCTCCTTGTTGC
GAPDH	Forward Primer	GTCTCCTCTGACTTCAACAGCG
	Reverse Primer	ACCACCCTGTTGCTGTAGCCAA

### In silico analysis

In silico analysis of selected markers was performed using gene expression profiles generated as part of The Cancer Genome Atlas (TCGA) Program of the National Cancer Institute. The gene expression data was generated through RNA-sequencing using an Illumina HiSeq2000 (Cancer Genome Atlas Network 2015). In this study, the TCGA dataset of Head and Neck Squamous Cell Carcinoma (TCGA-HNSC) was selected for further downstream analyses.

The comparison of the gene expression in NFE2L2, MAPK1, and MTOR across patients with and without survival was performed using the complete TCGA-HNSC data (*n* = 496). Further analyses on the genes NFE2L2, AKT1, ADRB2, MAPK1, and MTOR were performed on a selection of patients with an above-median ADRB2 expression (*n* = 248). Unpaired *t*-test (Mann–Whitney) was used to analyses differences between dual comparisons (alive vs. dead, recurrence vs. no recurrence), whereas the comparison of multiple groups (disease stage) was analyzed using Kruskal–Wallis analysis. Also, Kaplan-Meier curves were generated presenting overall survival in ADRB2-high patients in relation to selected genes. Patient groups were separated according to median expression and survival curves were analyzed using Mantel-Cox test. *P*-values below 0.05 were considered to be significant Analyses were performed using GraphPad Prism 8.

## Supplementary information

supplemetal figure 1

supplemetal figure 2

Supplementary figure legends
