# Supplementary figures and images for: β2-AR blockade potentiates MEK1/2 inhibitor effect on HNSCC by regulating the Nrf2-mediated defense mechanism
Source: Cell Death Dis. 2020 Oct 13;11(10):850. doi: 10.1038/s41419-020-03056-x (PMC7555890; doi:10.1038/s41419-020-03056-x)

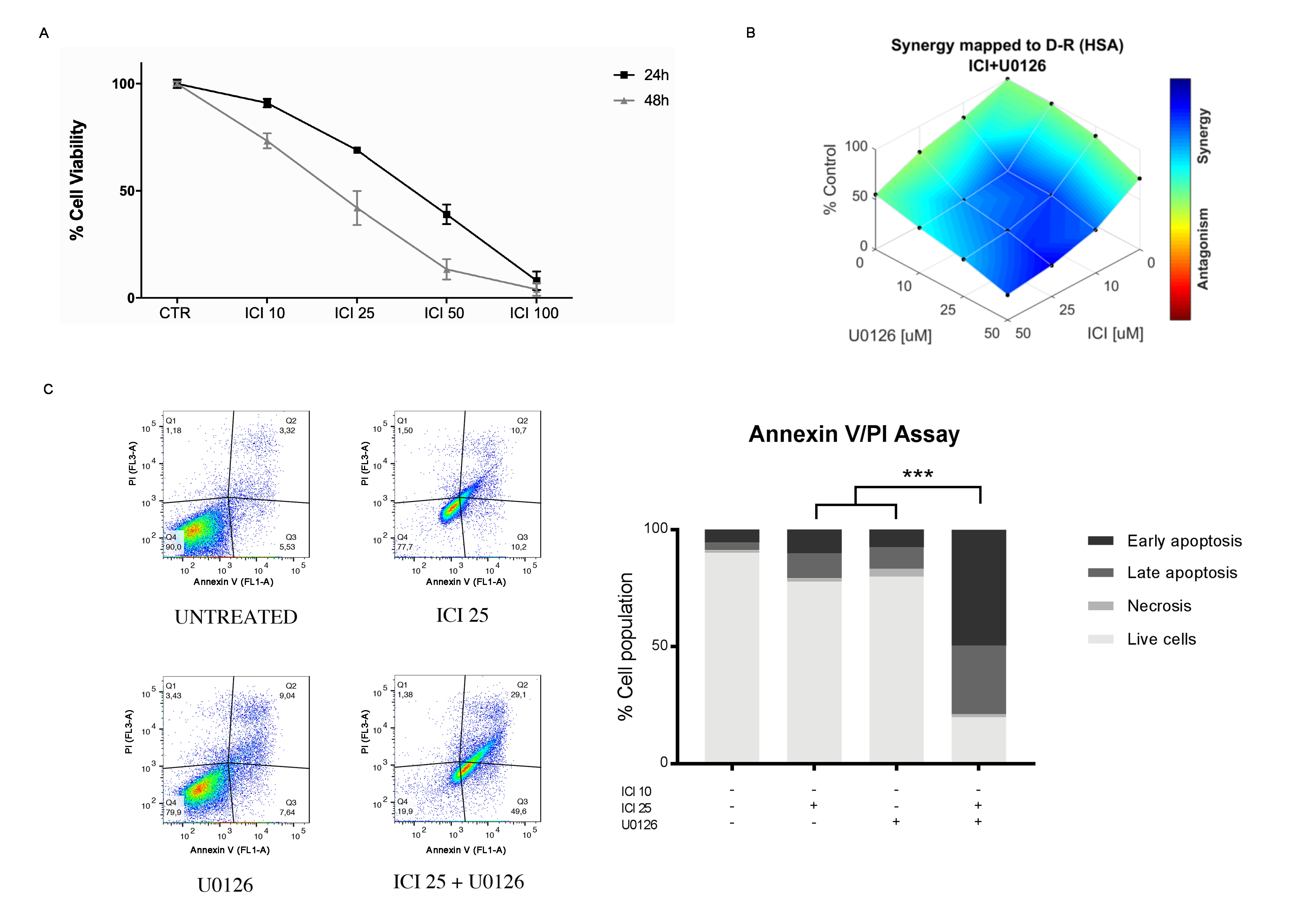

Supplement: Supplementary file 1 — supplemetal figure 1 [file 41419_2020_3056_MOESM1_ESM.png]

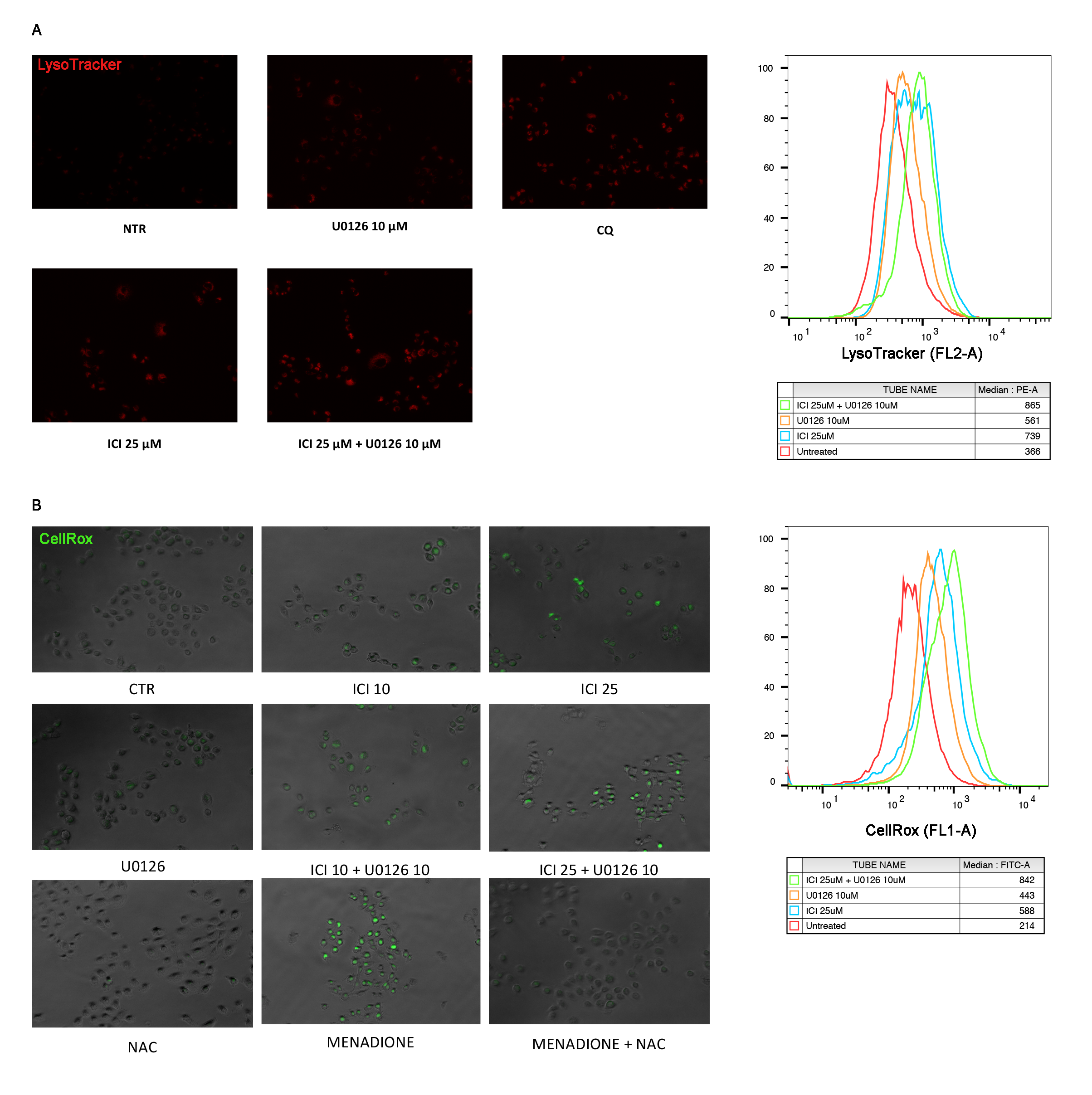

Supplement: Supplementary file 2 — supplemetal figure 2 [file 41419_2020_3056_MOESM2_ESM.png]
