# Supplementary material for: β2-AR blockade potentiates MEK1/2 inhibitor effect on HNSCC by regulating the Nrf2-mediated defense mechanism
Source: Cell Death Dis. 2020 Oct 13;11(10):850. doi: 10.1038/s41419-020-03056-x (PMC7555890; doi:10.1038/s41419-020-03056-x)
Supplement: Supplementary file 3 — Supplementary figure legends [file 41419_2020_3056_MOESM3_ESM.docx]

**Supplementary figure legends**

**Fig. S1**

β2-AR and MEK1/2 inhibitors increase CAL 33 cell death by apoptosis, in a synergistic way. **a** Viability assay on CAL 33 treated with ICI at 24 h and 48 h. The β2-AR inhibitor IC50 was ~40 μM at 24 h and ~20 μM at 48 h. **b** The values are represented in the combination index plot in function of ICI/U0126 tested concentrations. The drugs show a significant synergism in combinations. **c** Annexin V/PI flow cytometry analysis to determine apoptosis in ICI and U0126-treated cells. Drug combination promote a strong apoptosis effect, on CAL 33, compared to the single treatments (***P ≤ 0.001).

**Fig. S2**

β2-AR inhibition induces autophagy and oxidative stress. **a** IF and flow cytometry for LysoTracker. ICI treatment increase the number and size of lysosome on CAL 33, after 24 h. Flow cytometry histograms for the LysoTracker are shown in the graphs. **b** IF and Flow cytometry with CellROX (for oxidative stress determination), 24 h posttreatment (menadione is used as positive control; N-acetylcysteine (NAC) is used as ROS scavenger). ICI treatment on CAL 33 strongly increases ROS production, instead of U0126 which has a milder effect. The Flow Cytometry experiments have been performed in triplicates and the statistical analysis confirmed the significance of the results (*P ≤ 0.05).
